# SARS-COV-2 and Male Reproductive Health

**DOI:** 10.5935/1518-0557.20200047

**Published:** 2020

**Authors:** Renato Fraietta, Fábio Firmbach Pasqualotto, Matheus Roque, Paulo Franco Taitson

**Affiliations:** 1 Department of Surgery, Division of Urology, Universidade Federal de São Paulo, São Paulo, Brazil; 2 Department of Health Sciences, School of Medicine, University of Caxias do Sul, Caxias do Sul, RS, Brazil; 3 Department of Reproductive Medicine, Mater Prime, São Paulo, Brazil; 4 Discipline of Human Reproduction, Institute of Biological Sciences and Health, Pontifical Catholic University of Minas Gerais, Brazil

**Keywords:** Semen, Covid-19, SARS-COV-2, male reproductive

## Abstract

Critical challenges for the public and private health, research, and medical communities have been posed by the COVID-19 outbreak. Some of these challenges are related to the possible adverse effects of SARS-CoV-2 on male reproductive health, and whether other potential modes of transmission may occur, such as sexual transmission. Moreover, concerns have been raised in terms of whether the COVID-19 outbreak may have an impact on fertility worldwide. In this study, we will discuss the origins of SARS-CoV-2. We will further describe its mechanism of action, diagnosis, symptoms, and potential effects on the male reproductive system.

## Introduction

As of May 16, 2020, 4,434,653 confirmed cases and 302,169 deaths due to the novel coronavirus disease 2019 (COVID-19) - which is caused by the new, severe acute respiratory syndrome coronavirus 2 (SARS-CoV-2) - have been reported worldwide (WHO, 2020a). Although COVID-19 was first identified as a respiratory disease, it is now considered a systemic pathology, as it may affect different systems (Pan *et al.*, 2020). New challenges are now emerging concerning the disease, such as associated reproductive implications and the consequences of assisted and natural conceptions in the presence of acute COVID-19 infection and following patient recovery (Eisenberg 2020).

 In the last 20 years, there were two previous pandemics related to coronavirus, including the severe acute respiratory syndrome (SARS; 2002 and 2003) and the Middle East respiratory syndrome (MERS; 2012), which did not reach the number of cases and deaths associated with COVID-19. However, these previous pandemics have accelerated our understanding of the epidemiology and pathogenesis of SARS-CoV-2 (Jin *et al.*, 2020), and an unprecedented number of research studies related to this disease have emerged. Further, over 500 clinical trials have been registered among different national and international clinical trial registry sites as of April 21, 2020, which aim to evaluate different possible therapeutic options (Thorlund *et al.*, 2020). Nevertheless, until now, no specific and effective therapeutic strategies have been available to decrease the fatality rates of COVID-19, and it seems that quarantine, isolation, and social distancing remain the best strategies to deal with this novel pandemic (Hick & Biddinger, 2020). Thus, critical challenges for the public and private health, research, and medical communities have been posed by the COVID-19 outbreak (Fauci *et al.*, 2020). Some of these challenges are related to the possible adverse effects of SARS-CoV-2 on male reproductive health, and whether other potential modes of transmission may occur, such as sexual transmission. Moreover, concerns have been raised in terms of whether the COVID-19 outbreak may have an impact on fertility worldwide (Cardona Maya *et al.*, 2020).

In this study, we will discuss the origins of SARS-CoV-2. We will further describe its mechanism of action, diagnosis, symptoms, and potential effects on the male reproductive system.

## SARS-CoV-2 history and origin

On December 31, 2019, Chinese health authorities announced dozens of pneumonia infections of unknown etiology in Wuhan city (Hubei province). The infectious agent was identified on 7 January 2020, and was classified as a novel coronavirus (2019-nCoV)(Abduljalil & Abduljalil, 2020). On January 30, 2020, the World Health Organization (WHO) decreed a public health emergency of international importance (Souza *et al.*, 2020). Thereafter, on February 11, 2020, the WHO announced that this novel coronavirus pneumonia was classified as coronavirus disease-2019 (COVID-19)(WHO, 2020b). Due to its similarity with the causative agent, SARS, the novel coronavirus was named SARS-CoV-2 by the International Committee on Taxonomy of Viruses (Abduljalil & Abduljalil, 2020). Given the global involvement of COVID-19, the WHO declared a pandemic on March 11, 2020 (Souza *et al.*, 2020).

SARS-CoV-2 is the seventh identified member of the coronavirus family to affect humans, and it is the third coronavirus to emerge in the human population in the past two decades. Many details related to its origin and its ability to spread among humans remain unknown (Munster *et al.*, 2020). Coronaviruses are a large family of enveloped, single-stranded RNA viruses known to infect not only humans, but also other mammals and birds, leading to respiratory, hepatic, gastrointestinal, and neurologic diseases (Mungroo *et al.*, 2020; Zhu *et al.*, 2020). A genome-wide phylogenetic analysis indicated that SARS-CoV-2 shares 79.5% and 50% sequence identity with SARS-CoV and MERS-CoV, respectively (Jin *et al.*, 2020). Although believed to originate from bats, it has been speculated that other animals, such as pangolins and snakes, served as intermediate hosts, allowing the spillover of SARS-CoV-2 as a distinct human virus (Abduljalil & Abduljalil, 2020). However, phylogenetic analyses of the virus and its closely related reference genomes indicate that the origin of the virus has yet to be determined (Zhou *et al.*, 2020).

## SARS-CoV-2 mechanism of action

SARS-CoV-2 has four key structural proteins, namely nucleocapsid (N), spike (S), small membrane (SM), and membrane (M) proteins. The S protein is required for the virus to fuse to the host cell through the receptor-binding-domain (Monteleone *et al.*, 2020). The main path for SARS-CoV-2 entry into the cell is via the attachment of the S protein to the angiotensin-converting enzyme 2 (ACE2) that can be identified in type II alveolar cells, myocardial cells, proximal tubule cells of the kidney, ileum and esophagus epithelial cells, and bladder urothelial cells (Zhou *et al.*, 2020). ACE2 can also be identified in the human testis, as it is highly expressed in Leydig and seminiferous tubules cells (Fan *et al.*, 2020). Thus, it is hypothesized that SARS-CoV-2 may bind to ACE2 in testicular tissue, leading to alterations in testicular tissue and providing a site for viral infection (Cardona Maya *et al.*, 2020) Following membrane fusion, viral RNA is released into the cytoplasm, and viral replication begins (Monteleone *et al.*, 2020).

It has been considered that the main path for SARS-Cov-2 transmission is from person to person through droplets and close contact (Chan *et al.*, 2020). However, it has also been hypothesized that other transmission routes may be available, although they require further verification (Uddin *et al.*, 2020; Halfmann *et al.*, 2020). In reproductive medicine, special consideration has been paid as to whether vertical transmission (mother-to-fetus)(Simões e Silva *et al.*, 2020) and sexual transmission may occur (Li *et al.*, 2020).

## COVID-19 diagnosis and symptoms

The most frequently occurring symptoms of COVID-19 include fever, cough, fatigue, shortness of breath, sputum production, headache, and myalgias. Patients may also complain of vomiting, diarrhea, anosmia, and also ophthalmologic and cutaneous manifestations (Segars *et al.*, 2020). Interestingly, in a recently published study evaluating the presence of the coronavirus in the semen of infected patients, it was noted that although no SARS-CoV-2 was identified in semen samples, ≈18% of infected men reported scrotal discomfort at the time of COVID-19 infection (Pan *et al.*, 2020).

COVID-19 patients have been classified as asymptomatic, mild, severe, and critical types. Mild patients tend to experience mild pneumonia, while severe patients exhibit dyspnea and increased respiratory frequency within 24-48 hours. Critical patients suffer from respiratory failure, acute heart injury, septic shock, and multiple organ failure (Mungroo *et al.*, 2020).

SARS-CoV-2 is a highly pathogenic virus that may be associated with uncontrolled cytokine release, known as a cytokine storm, that may lead to capillary leakage, tissue toxicity, edema, organ failure, and shock (Zhou *et al.* 2020). In COVID-19, a significant elevation of cytokines (interferon [IFN]-γ, tumor necrosis factor [TNF]-α, interleukin [IL]-6, IL-10, IL-2, IL-1, and others) and lymphocytopenia are found. The clinical manifestation of the severe type in patients is significantly related to elevated IL-6 (Zhou *et al.*, 2020).

Although the presenting symptoms and radiographic analysis of computed tomography (CT) scans may suggest the presence of COVID-19, a definitive diagnosis is achieved through virus detection via polymerase chain reaction (PCR). It is recommended by the U.S. Centers for Disease Prevention and Control (CDC) that a PCR is performed to diagnose acute infection. False-positive SARS-CoV-2 testing is rare, although false-negative results can occur due to inadequate sample collection or if it is performed early in the disease course. PCR may be performed with samples obtained from nasal swabs, trachea and nasopharynx extracts, and several primers used to detect SARS-CoV-2, which were established through real-time reverse-transcription (RT)-PCR. This enables fast and specific virus detection (Mungro *et al.*, 2020; ASRM COVID Task Force, 2020). Serologic tests, evaluating the presence of immunoglobulin M (IgM) and immunoglobulin G (IgG) antibodies are not recommended for diagnosis of acute infection, although emerging evidence suggests that they may confer immunity or reduced risk of reinfection. When performed by ELISA, serologic tests present >95% specificity for disease diagnosis, but sensitivity may range from 60%-98% (ASRM COVID Task Force, 2020).

## SARS-CoV-2 and the male reproductive tract

- **Does the virus affect the male reproductive tract?**

Until now, it is unknown whether or to what extent SARS-CoV-2 can affect male reproductive health (Wang & Xu, 2020; Esteves *et al.*, 2020; Stanley *et al.*, 2020). COVID-19 is primarily contracted through droplets; however, the virus has already been isolated in the urine (Guan *et al.*, 2020), feces (Guan *et al.*, 2020), and conjunctiva (Xia *et al.* 2020) of infected patients. Due to its mechanism of action - i.e., ACE2 receptor binding - it may compromise other tissues with ACE2 receptors, such as those of the reproductive system. Specifically, high ACE2 expression levels are found in testicular cells, mainly in seminiferous duct cells, spermatogonia, and Leydig and Sertoli cells (Esteves *et al.*, 2020).

**• Testicle**

Studies of SARS-CoV reveal orchitis as a possible clinical presentation of this virus, and there is evidence of deleterious effects on testicular tissues, including the presence of the virus on autopsy (Xu *et al.*, 2006; Zhao *et al.*, 2003). As in SARS-CoV, the ACE2 receptor plays an important role in the pathophysiology of SARS-CoV-2 infection, as it is used as the primary form of cell binding, leading the virus to infect the cell and replicate. Several studies have demonstrated a high concentration of ACE2 in testicular tissues, either in germ cells or somatic cells (Fan *et al.*, 2020; Wang & Xu, 2020; Shen *et al.*, 2020; Zhang *et al.*, 2020). Thus, there is evidence that the testis is vulnerable to SARS-CoV-2 infection, so it is important to assess and monitor the reproductive functions of these patients. In addition, the presence of orchitis complaints was found in 19% of patients (Pan *et al.*, 2020). However, a recent study evaluated patients with COVID-19 and showed a complete absence of SARS-COV-2 in the semen and testes of infected men (Song *et al.*, 2020). Therefore, more studies examining more patients are needed to confirm whether the virus is present in the testes.

**• Prostate**

Only one small retrospective study evaluated the presence of SARS-CoV-2 in prostatic secretion (PS). A Chinese study evaluated the PS of 18 males diagnosed with COVID-19 and five suspected cases. The samples of all evaluated patients did not show evidence of the RNA expression of SARS-CoV-2 (Quan *et al.*, 2020).

**• Seminal sample**

Although there is a protective blood-testicular barrier, more than 27 viruses can be found and transmitted through the semen, such as human immunodeficiency virus (HIV), mumps, influenza, Zika virus, coxsackievirus infection, Ebola, and hepatitis B and C. Two previous studies did not find SARS-CoV-2 in semen; however, these studies had low numbers of patients who were at different stages of infection and recovery (Pan *et al.*, 2020; Song *et al.*, 2020). The first study to evaluate the semen of males diagnosed with COVID-19 evaluated 34 Chinese males. Although six (19%) of these patients complained of scrotal discomfort due to viral orchitis, SARS-CoV-2 was not detected in the semen of any of these patients (Pan *et al.*, 2020). The second study evaluated 12 patients in the recovery phase of COVID-19, and the semen evaluation of all patients showed no detectable SARS-CoV-2 RNA in the semen samples (Song *et al.*, 2020).

The third study published evaluating the semen samples of men with COVID-19 featured 38 patients. The study found that in six (15.8%) of these patients, SARS-CoV-2 was detected in the semen samples, even among those who were recovering. However, the study was not able to evaluate virus shedding, survival time, and viral concentration in semen. This study raises question of whether SARS-CoV-2 can be sexually transmitted, as this might represent a critical factor in transmission prevention (Li *et al.*, 2020). Thus, as of May 15, 2020, data are available on the semen samples of 84 male patients with COVID-19, and it was found that SARS-CoV-2 was identified in six (7.1%).

There are no data in the literature regarding changes in the fertile potential of these men affected by SARS-CoV-2, although it is known that any feverish condition is capable of altering seminal quality (Carlsen *et al.*, 2003; Jung & Schuppe, 2007). However, it is important to assess whether there are any such direct effects associated with this virus, as occurs in cases of mumps infection (Davis *et al.*, 2010).

**• Hormonal dosages**

There was an evaluation of gonadal function in some patients, which was achieved through hormone profile measurement. When compared to healthy patients, those infected showed a probable initial gonadotoxic effect (Zhang *et al.*, 2020). However, more data are necessary to confirm the gonadotoxic effects of the virus.

## Conclusion

There is the theoretical possibility that testicular damage and subsequent infertility may result following COVID-19 infection, and also the possibility of sexual transmission, as SARS-CoV-2 has been identified in the semen of infected patients. However, the available data and study findings are recent, based on small sample sizes, and present conflicting information. Thus, until now, there is not enough evidence to support the need for asymptomatic couples to avoid sexual intercourse to protect against virus transmission. Further research is needed to understand the long-term impacts of SARS-CoV-2 on male reproductive function, including its potential effects on fertility and testicular endocrine function. Before arriving at a definitive understanding of the impacts of potential viral attacks on the testis, more detailed physiological and pathological examinations of the male reproductive systems of COVID-19 patients after their recovery are needed.
